# Air Quality Awareness Week and Asthma Awareness Month — May 2014

**Published:** 2014-04-25

CDC is collaborating with the U.S. Environmental Protection Agency to urge U.S. residents to pay attention to their local air quality during Air Quality Awareness Week, April 28–May 2, 2014. May also is Asthma Awareness Month, and May 6 is World Asthma Day.

Asthma sufferers are particularly affected by air pollution. One in 12 U.S. residents (approximately 25.5 million persons) currently has asthma, and nine persons in the United States die from asthma-related complications every day (1). Ozone air pollution, more common in the summer months, can trigger asthma attacks, leading to increased medication use, visits to emergency departments, and hospital admissions. Persons with asthma and other at-risk groups can use daily forecasts of the Air Quality Index to plan exercise and other outdoor activities for times when air pollution is predicted to be low.

Persons with asthma and other chronic lung diseases, such as emphysema and chronic bronchitis, are not the only ones affected by ozone. Children, older adults, and active persons of all ages who exercise or work vigorously outdoors also are at risk. Ozone can irritate the respiratory system, reduce lung function, and inflame and damage the lungs. Over time, ozone exposure can cause permanent lung damage.

Daily air quality forecasts and current conditions for 400 U.S. cities are available at http://www.airnow.gov and through the AirNow mobile app (http://m.epa.gov/apps/airnow.html) and Enviroflash e-mail service (http://www.enviroflash.info).

Information on Air Quality Awareness Week is available at http://epa.gov/airnow/airaware/index.html. Information on Asthma Awareness Month is available at http://www.epa.gov/asthma/awareness.html. Additional information about asthma is available from CDC at http://www.cdc.gov/asthma.
